# Mobile Therapeutic Attention for Treatment-Resistant Schizophrenia (m-RESIST) Solution for Improving Clinical and Functional Outcomes in Treatment-Resistant Schizophrenia: Prospective, Multicenter Efficacy Study

**DOI:** 10.2196/67659

**Published:** 2025-05-15

**Authors:** Jussi Seppälä, Eva Grasa, Anna Alonso-Solis, Alexandra Roldan-Bejarano, Marianne Haapea, Matti Isohanni, Jouko Miettunen, Johanna Caro Mendivelso, Cari Almazán, Katya Rubinstein, Asaf Caspi, Zolt Unoka, Kinga Farkas, Elisenda Reixach, Jesus Berdun, Judith Usall, Susana Ochoa, Iluminada Corripio, Erika Jääskeläinen, m-Resist Group, Francisco Alcalde

**Affiliations:** 1 Social Insurance Institution of Finland, Suokatu 40, Kuopio, 50700, Finland, 358 0413172293; 2 Research Unit of Population Health, University of Oulu, Oulu, Finland; 3 Mental Health, Institut de Recerca Sant Pau IR SANT PAU, SantQuintí 77-79, Barcelona, Spain; 4 Centro de Investigación Biomédica en Red de Salud Mental CIBERSAM, Madrid, Spain; 5 Mental Health Division, Fundació Althaia, Xarxa Assistencial Universitaria de Manresa, Manresa, Spain; 6 Medical Research Center Oulu, Oulu University Hospital and University of Oulu, Oulu, Finland; 7 Department of Psychiatry, Oulu University Hospital, Oulu, Finland; 8 Agency for Health Quality and Assessment of Catalonia AQuAS, Barcelona, Spain; 9 The Gertner Institute of Epidemiology and Health Policy Research, Sheba Medical Center, Tel Aviv University, Tel Aviv, Israel; 10 Department of Psychiatry and Psychotherapy, Semmelweis University, Budapest, Hungary; 11 TicSalut Health Department, Generalitat de Catalunya, Barcelona, Spain; 12 Digital Health Unit, Hospital de la Santa Creu i Sant Pau, Institut d’Investigació Biomèdica Sant Pau IIB SANT PAU, Barcelona, Spain; 13 Parc Sanitari Sant Joan de Déu, Sant Boi de Llobregat, Spain; 14 Etiopatogènia i tractament dels trastorns mentals greus MERITT, Institut de Recerca Sant Joan de Déu, Esplugues de Llobregat, Spain; 15 Mental Health and Psychiatry Department, Vic Hospital Consortium, Vic, Spain; 16 Mental Health, Institut de Recerca Sant Pau IR SANT PAU, Sant Quintí 77-79, Barcelona, 08041, Spain; 17 Unit of Population Health, University of Oulu, Oulu, Finland

**Keywords:** digital health, mHealth, m-RESIST, application, treatment-resistant schizophrenia, psychosis, feasibility study, mobile phone, mobile health, mobile therapeutic attention for treatment-resistant schizophrenia

## Abstract

**Background:**

Treatment-resistant schizophrenia (TRS) is a severe form of schizophrenia associated with low adherence to treatment and poor outcomes. Mobile health (mHealth) interventions may be effective in preventing relapses, increasing treatment adherence, and managing some of the symptoms of schizophrenia. Mobile therapeutic attention for treatment-resistant schizophrenia (m-RESIST) is an innovative mHealth developed specifically for TRS.

**Objective:**

We aim to evaluate the effects of m-RESIST on the clinical and functional outcomes and on the perceived quality of life in people with TRS.

**Methods:**

A feasibility study without a control group was performed to test the m-RESIST solution on patients with TRS. Participants were recruited from Spain, Israel, and Hungary. This study’s population (N=31) followed 3 months of intervention. The m-RESIST was configured by an app, a wearable, and a web-based platform. The severity of symptoms was evaluated by using the Positive and Negative Syndrome Scale (PANSS) and the Clinical Global Impression-Schizophrenia (CGI-SCH) scale. Functionality was assessed by the Global Assessment of Functioning and perceived quality of life was evaluated by the EuroQol visual analogue scale (EQ-VAS).

**Results:**

Significant reductions were found in symptoms from pretrial to posttrial on the PANSS total (mean difference −7.2, 95% CI −11.1 to −3.4; *P*=.001), the PANSS positive (mean difference −1.36, 95% CI −2.6 to −0.1; *P*=.04), the PANSS negative (mean difference −2.1, 95% CI −3.1 to −1.1; *P*<.001), and the PANSS general symptoms (mean difference −3.8, 95% CI −6.8 to −0.8; *P*=.02). In almost one-fifth of the participants (6/31), the overall score for the PANSS decreased by more than 20%, which may be considered a clinically significant change. On the CGI-SCH scale, the sum of total severity of illness decreased significantly (*P=.*03). A decrease in the sum of positive and negative symptoms of the CGI-SCH score was also found (*P=.*04 and *P=.*03, respectively). The sum of depressive or cognitive symptoms did not change. The functionality of participants increased significantly on the Global Assessment of Functioning (*P*≤.001). The perceived quality of life on the EQ-VAS also improved (mean difference 6.7, 95% CI 0.5 to 12.9; *P=.*04).

**Conclusions:**

To our best knowledge, this was the first study to address the efficacy of the mHealth app m-RESIST on the symptoms and functional capacity and on the quality of life for people with TRS. Our preliminary findings showed that implementing the m-RESIST solution decreased the symptoms and severity of disease, and improved the functionality and perceived quality of life among those with TRS. The change of symptoms on the PANSS total may be clinically significant. Modern technologies such as mHealth interventions may be useful in treating symptoms and functionality even in TRS, which is a major clinical challenge, with usually poor outcomes. These results should be corroborated by performing a controlled trial.

## Introduction

### Background

Schizophrenia is a severe mental disorder and has a prevalence of 1% of the population worldwide [1]. A meta-analysis showed that only 13.5% of the patients were in recovery, which included clinical remission and good social functioning outcome [2]. Despite the common use of antipsychotic drugs, 30%‐50% of patients with schizophrenia still obtained a low benefit from conventional treatments [3,4]. This inadequate response to pharmacological treatment is known as treatment-resistant schizophrenia (TRS). TRS implicates an important burden at three levels, as follows: (1) clinical (negative attitude to medication, drug abuse, and nutritional or physical health problems); (2) economic (hospitalizations and polytherapy); and (3) humanistic (depression and social isolation) [5]. As a result, dealing with TRS involves a high emotional burden for patients and their caregivers, affecting their quality of life. The current health care and social support systems are unable to provide adequate and effective solutions to these patients.

Results from earlier studies on psychosis [6-8] supported that information and communication technology (ICT) tools in the treatment of patients with TRS would be useful. Smartphone ownership among people with schizophrenia is also relatively high and increasing [9-11]. Patients seemed capable of using mobile phones to monitor their symptoms, participate in therapeutic interventions, and increase physical exercise [12]. Previous feasibility studies have shown that interventions based on mobile health (mHealth) could promote the empowerment of patients with schizophrenia [9,13]. Some mHealth interventions may also be effective for increasing treatment adherence and decreasing relapses [13,14].

Several digital solutions also specifically for schizophrenia exist [15,16]. Apps such as FOCUS [6,17-19], SMARTapp [20], MACS [21], weCope [22], My Journey 3 [23,24], and App4Independence [25] offer disease management strategies in general. Others are more focused on symptom monitoring, namely Crosscheck [26], Actissist [27], ClinTouch [28], ExPRESS [29], Ginger.io [30], ReMindCare [31], and MindFrame [32]. Other mobile apps are supported in cognitive behavioral therapy (SlowMo) [33]; TechCareApp [34]; movisensXS [35]; PRIME [36], or work as an agenda (MONEO) [37], or target specific domains such as social skills (MASS) [38], sleep (ExpiWell) [39], medication adherence (MedActive) [40], or delivers positive psychology content (+ Connect) [41]. Digitally delivered psychological therapies, the use of social media, virtual reality, and chatbots are also digital solutions that may have a role in the treatment of those with schizophrenia [42,43]. Digital phenotyping and machine learning, virtual reality interventions, and language models, with ChatGPT, as a well-known example, have developed in recent years [42,43].

A systematic review and meta-analysis by Vitger et al [44] examined digital shared decision-making interventions in mental health care, analyzing 16 randomized controlled trials (RCTs), of which only 3 evaluated apps. The findings suggest that digital shared decision-making tools may positively impact patient activation, decisional conflict, working alliance, and symptom severity. Specific digital interventions include a tablet app for patients with schizophrenia, designed to assess and record satisfaction with life and treatment domains, facilitating discussions between patients and nurses [45]. Another tablet app addresses concerns raised by patients with psychosis, providing methods for clinicians and patients to collaboratively explore and manage issues [46]. Additionally, the smartphone app “Power Up” aims to promote patient activation among children and adolescents facing mental health challenges [47].

Patient-reported outcomes collect data directly from patients. These data are used in clinical practice, helping decision-making. A systematic mapping review analyzed the characteristics and outcomes of mHealth interventions in psychosis based on 29 publications reporting on 23 studies [48]. Objective measures included data passively obtained from phone usage or embedded sensors. Subjective measurement instruments include either observer-rated or self-report questionnaires that can be written or administered digitally, whether regarding their health status or experience using the mHealth intervention [48]. Outcomes were grouped into 8 categories, namely severity of psychosis-related symptoms, functioning, well-being, medication adherence, adverse events, user experience, technical, and all other outcomes reported [48].

Real-time and in-context patient-generated symptom data, obtained through remote-monitoring platform technology, have the potential to timely warn clinicians about the need for intervention, improve treatment decisions by providing a clearer picture of changing patterns of symptoms, and support scheduling of health care contacts based on need [42].

Systematic comparison of apps with other digital interventions may help us to better understand their roles in mental health care. The main use of smartphone data (active and passive) is tracking mood and lifestyle in people with major depression, bipolar disorder, and psychosis. They also have future potential in machine learning toward individualized risk prediction and delivery of targeted “just in time” intervention. Key issues are a lack of validation across studies and establishing trust around data usage. Priority actions include data standards for interoperability and validation, and industry-academic partnerships around access [42].

Digitally delivered psychological therapies are used in the self-management of symptoms of depression and anxiety. They have the potential for precision interventions and preventive treatments. Lack of engagement, saturated consumer marketplace, and claims outpacing clinical evidence are major issues. Establishing an evidence base for use in people with diagnosed mental disorders is warranted [42].

Population level monitoring of mood and anxiety is possible with the help of social media. The future potential of social media lies on its potential for real time monitoring of mental health state and for access to peer support. Key issues are sampling bias, access to data from social media companies, and also privacy. Priority actions are industry-academic partnerships and privacy standards [42].

The main use of virtual reality is exposure therapies, and it may have higher engagement and potentially higher efficacy than apps. Increased accessibility, low-cost headsets, and expanded clinical targets are important [42]. Chatbots have future potential to increase access to care but they have limited range of appropriate response. Establishing evidence base for use in people with diagnosed mental disorders is needed [42].

The 5 principles of the Collaborative Care Model are evidence-based care, measurement-based treatment to target, population health management, accountable care, and team-based care [49]. Practices with integrated mental health clinicians can maximize the impact of digital mental health tools by integrating digital information and interventions into the course of clinical care. This allows for the practice of hybrid care, a combination of synchronous and asynchronous health care, which can enable increased access to care. Hybrid care can address the known challenge of low retention in digital health tools, given that a digital navigator or clinician can promote engagement, guide the patient to relevant content, and, depending on the tool, use data from the patient’s use in further guiding face-to-face care [49].

The findings from the pilot study suggest that smartphone apps may become a useful tool for psychiatric rehabilitation, addressing both psychiatric and co-occurring medical problems. Individualizing functions to each patient and facilitating connection with a certified peer specialist may be an important feature of useful apps [50].

Apps can be successfully integrated into real-world care [43]. These models of care involve both synchronous (eg, a therapy visit) and asynchronous telehealth (ie, app use between visits) and can provide therapeutic synergy between in-person and remote care [51]. The ReMindCare app for early psychosis has been integrated into clinics since 2018, and implementation models for integrating apps in youth services for schizophrenia, and coordinated specialty, have already been developed [31,52-54]. While the actual dissemination of these models is impossible to ascertain, they can already support the use of apps to improve vocational training, educational opportunities, medication adherence, cognitive training, symptom tracking, psychoeducation, and more. Apps for schizophrenia have also led to the development of human support roles as part of mental health services to facilitate their implementation in clinical practice, such as the FOCUS mHealth support specialist [55] and the mindLAMP digital navigator [53].

A renewed focus on privacy and data protection is essential for the success of mental health apps, as users must trust these apps for widespread adoption. Many health-related apps collect sensitive data, including demographic, medical, and lifestyle information, sometimes even unrelated to the app’s intended purpose [42,43,56,57]. Despite the importance of privacy, a significant portion of mental health apps (31%‐49%) lack a privacy policy, and collected data can be shared with third parties or used for advertising [42,43,56,57]. Vulnerable populations, such as individuals with schizophrenia, require additional research protections to ensure their privacy and security [43].

A cross-cultural psychological assessment focuses on understanding how a client’s cultural background influences their behavior, emotional state, and psychological needs. A recent review of 2248 studies found that only 57 specifically targeted migrants, refugees, or cultural minorities [58]. Of these, half used digital health technologies developed for the general population without cultural adaptations, while the other half tailored the interventions to meet the specific needs of these populations [58]. However, only a small number of studies involved a high level of participation from these groups in the development and testing of the digital tools [58].

Despite rapid progress, there is room for improvement. Presently, there are fewer than 10 apps whose design was specifically tailored to people with schizophrenia and that are accessible for immediate public download on the Apple or Android (Open Handset Alliance [led by Google]) marketplaces. Moreover, many of these 10 apps appear neglected by developers after their launch, with an average time since the last update of 1121 days [43,59]. The number of studies on the efficacy of mHealth apps on the symptoms and functional capacity, and on the quality of life in schizophrenia and related psychotic disorders is limited according to recent systematic reviews [15,56,60,61].

The systematic review by Miralles et al [56] found 3 studies evaluating efficacy of mHealth solutions in schizophrenia spectrum and in other psychotic disorders. Two of the three papers found decrease in symptoms measured by the Positive and Negative Syndrome Scale (PANSS) [6,27]. The third one reported no significant change in the symptoms on the PANSS, or no positive changes in functioning and in quality of life [36]. .

Additionally, a cross-sectional study on WeChat use and endorsement of WeChat-based mHealth among people living with schizophrenia (N=400) in China was carried out. Compared to nonusers, WeChat use was associated with improved health outcomes, including lower psychiatric symptoms, lower depression, higher functioning, better recovery, and higher quality of life [62].

A recent study of patients with a diagnosis of schizophrenia, randomized to receive either SlowMo*,* a blended digital therapy targeting reasoning, or usual care, found no significant difference between groups on the primary outcomes related to paranoia at 24 weeks (the primary end‐point) [33].

### The Mobile Therapeutic Attention for Treatment-Resistant Schizophrenia Project

The project of mobile therapeutic attention for treatment-resistant schizophrenia (m-RESIST), funded by the Horizon 2020 Programme of the European Union, designed and developed a mobile therapeutic attention for TRS. This digital solution consisted of a new and innovative mHealth solution based on novel technology and offering highly modular and flexible functioning. This solution was developed specifically for patients with TRS and their caregivers based on their needs [63-65].

The positive acceptance of the m-RESIST solution was related to its usefulness in meeting user needs, its capacity to empower them, and its possibility of maintaining human contact [63].

The only mHealth study performed in those with TRS indicated that the m-RESIST solution is feasible and is an acceptable, satisfactory, and potentially useful tool for a population with TRS [65].

### Aims

In this study, our aim was to evaluate the effects of the m-RESIST solution on the clinical and functional outcomes and the perceived quality of life for those with TRS. To our best knowledge, there are no previous studies which have addressed these issues.

## Methods

### Study Design and Setting

This was a prospective, 3-month long, multicenter follow-up study without a control group that was carried out with patients with a diagnosis of TRS between March and November 2017. Blinding was not considered due to the absence of a comparator group. No sample size calculation was done.

In this study, the intervention involved patients and their caregivers, and the main actors involved in the deployment of the m-RESIST solution were psychiatrists, psychologists, and case managers. The key aim of the m-RESIST solution was to engage patients with TRS, together with their caregivers, in therapeutic processes, and empower them to enable their self-management.

Participants were recruited from 3 clinical sites: Sant Pau Hospital, Semmelweis University, Sheba Medical Center, and Gertner Institute of Epidemiology and Health Policy Research. These sites provided full-spectrum mental health services to adult people from their catchment area and have extensive experience in participating in large-scale clinical trials in patients with schizophrenia.

Patients underwent a 3-month long modular intervention designed for patients with TRS. The intervention was based on the recognition of early warning signs of psychosis to improve positive symptoms, treatment adherence, and a healthy lifestyle.

### The m-RESIST Solution

The intervention was supported by 3 mHealth tools: a wearable (smartwatch), a mobile app, and a web-based platform, which are presented in the figure of the protocol article. Data for usage was captured automatically by the m-RESIST software [64].

The functionality of the smartphone was based on the m-RESIST app. Through this app, patients had access to educational content about TRS condition and related issues; could track their early warning signs symptoms and biological variables (eg, sleep and steps counter); could ask for help with questionnaires or the “alarm button”; receive and practice helpful CBT-based coping strategies and exchange messages with their caregiver or health care provider [64].

The wearable was a smartwatch that collected data from patients and sent it wirelessly to the smartphone. Sensor data was recorded through automatic passive upload. The variables collected were the level of activity, heart rate, sleeping pattern, and steps counter [64].

The web-based platform was the tool that the health care providers (case manager, psychiatrist, and psychologist) used to collect assessment data, to monitor patients’ state and review data collected by sensors, to communicate by texting with patients, caregivers, and other professionals, and to consult recommendations (based on guidelines and experts’ opinion) [64]. Mental health care professionals helped patients with TRS in using the intervention [64]. The m-RESIST intervention was provided in 5 languages: Spanish, Catalan, Hungarian, Hebrew, and English.

The trial was registered in Clinical Trials (NCT03064776), and the study protocol was published earlier [64].

### Staff or Participant Training Protocols

Three user guides were prepared to ensure that all m-RESIST users (patients, caregivers, and clinicians) will be able to understand and follow the instructions of the devices or system used in home or health care facilities. The dashboard user guide explained quickly and simply the applications and functionalities that clinicians can perform when logging in. The mobile application installation guide was addressed to clinicians in case they need to install the m-RESIST solution in patients’ devices. This guide details the steps to download the m-RESIST application and install it on the smartphone and the smartwatch. The mobile application user guide was addressed to patients, caregivers, and clinicians and described the functionalities of the m-RESIST app working together with the smartwatch, once the app was installed on the Android smartphone provided. This user guide also specifies briefly the operations and the use of the smartwatch given to the patient at the beginning of this study. Finally, a short instruction sheet was designed. This sheet contained the main steps to charge smartphone and smartwatch devices and the main considerations to take into account to ensure devices were connected and worked properly.

In case of the PANSS, raters at each site had to perform a training to ensure liability between sites.

### Language Adaptation Process

To ensure the language adaptation process, the clinical research team from each site was responsible for adapting the m-RESIST solution from English to Spanish, Catalan, Hungarian, and Hebrew.

### Technical Support Procedures

To solve and improve the issues of the m-RESIST solution during the pilots, incidences were collected by the clinicians and communicated by email to the technical team. Thus, they could fix them.

### Ethical Considerations

This study protocol received approval from the ethics committees of the 3 participating institutions: Hospital Santa Creu i Sant Committee (IIBSP-RES-2016‐51), Egészségügyi Tudományos Tanács Tukeb, Semmelweis Ethical Committee (54920-4/2016/EKU), and Sheba Medical Center Ethical Committee (3472‐16 SMC). Participants were fully informed of the purpose and procedures of this study, and signed informed consent forms were obtained before any study-related activities were performed. The database generated by this study did not contain any identification of the participants but kept only a numerical code in a separate list; thus, the patients’ identities were protected. The information collected in this study was always treated as grouped data and never as individual or personal data, thereby maintaining anonymity and confidentiality. No compensation was provided to the participants.

### Study Participants

Patients with TRS were recruited for this study. The eligibility criteria were (1) patients aged 18‐45 years with a diagnosis of schizophrenia according to *Diagnostic and Statistical Manual of Mental Disorders, Fifth Edition* criteria; (2) duration of disease less than 15 years to meet the criteria for TRS [66,67]; (3) familiarity with ICT tools; and (4) physical capability to use them; and presence of a caregiver. Exclusion criteria were (1) remission according to the Remission of Schizophrenia Working Group [68]; (2) presence of delusions mainly related to their therapists or with new technologies; (3) presence of vision, hearing, or motor impairment, interfering with operating a smartphone; (4) presence of a caregiver or informal carer who is not used to ICT tools or has physical incapability to use them; and (5) presence of intellectual developmental disability. The caregivers were also recruited as part of the m-RESIST project.

A total of 81 patients with TRS, with their caregivers, were selected for invitation to participate by researchers. Almost half of them (n=39) were willing to enroll, and nearly 80% (n=31) of those started this study and followed the whole 3 months of intervention. The dropout rate was 18% (n=8).

### Measures in This Study

All the outcomes of this study’s protocol are shown in Table 1.

**Table 1.** Schedule of study protocol periods and assessments.

	Recruitment	Preintervention	Intervention						Follow-up
Activity or assessment		V0	V1	V2	V3	V4	V5	V6	V7
Eligibility screen	✓								
Informed consent	✓								
Delivery and training of devices	✓								
Sociodemographic data		✓							
Clinical characteristics		✓							
Relapse signature and treatment plan		✓							
Willingness to enroll	✓								
PANSS^a^		✓							✓
CGI-SCH^b^		✓	✓	✓	✓	✓	✓	✓	✓
GAF^c^		✓							✓
EQ-VAS^d^		✓							✓

a PANSS: Positive and Negative Syndrome Scale.

b CGI-SCH: Clinical Global Impression Schizophrenia.

c GAF: Global Assessment of Functioning.

d EQ-VAS: EuroQol visual analogue scale.

In this study, the severity of symptoms was assessed using the PANSS and the Clinical Global Impression-Schizophrenia (CGI-SCH) scale [69,70].

- The PANSS was used to assess positive and negative symptoms of schizophrenia. This scale consists of 30 items rated from 1 to 7 according to increasing levels of psychopathology, where: 1=absent, 7=extreme. Four scores are obtained: positive, negative, general symptoms, and total. The PANSS was completed on 2 occasions, at the start (VO) and the end (V7) of the intervention.
- The CGI-SCH scale measures global clinical impression and consists of 5 different ratings (positive, negative, depressive, cognitive, and global) that are rated from 1 to 7, where: 1=normal, 7=among the most extremely ill patients. the CGI-SCH scale was evaluated in all protocol visits (V0-V7).

### Functional and Perceived Quality of Life Outcomes

Functionality was assessed by using the Global Assessment of Functioning (GAF) scale [71] and perceived quality of life by the EuroQol visual analogue scale (EQ-VAS) [72].

- The GAF scale ranges from 1 (maximum level of disease) to 100 (the healthiest). It was performed on 2 occasions, at VO and at V7.
- The EQ-VAS is scored on a 20 cm visual analogue scale with end points labeled “the best health you can imagine” and “the worst health you can imagine.” It was administered on 2 occasions, at V0 and V7.

### Adverse Events

Clinician asked for adverse events at each pilot visit, and they were reported. Safety measures were collected throughout this study. The presence of serious and nonserious adverse events, defined as any clinical change or illness reported during this study, was monitored in every clinical visit. The adverse events observed when carrying out this study, either by the clinician or by the patient themselves and regardless of the causality relationship ascribed, were recorded in the clinical records and on the patient`s dashboard.

### Statistical Analysis

The sociodemographic characteristics of the participants with TRS were presented using frequency distributions for categorical variables and mean with SD for age. The outcomes in baseline and follow-up were presented using mean with SD for PANSS scores and EQ-5D, and medians with IQR for CGI-SCH and GAF scores. The change from the baseline to the follow-up was analyzed using the paired *t* test (PANSS scores and EQ-5D) or the Wilcoxon test (CGI-SCH and GAF scores). Statistical analyses were performed using the SPSS Statistics (version 29.0; IBM Corp).

## Results

**Figure 1.**
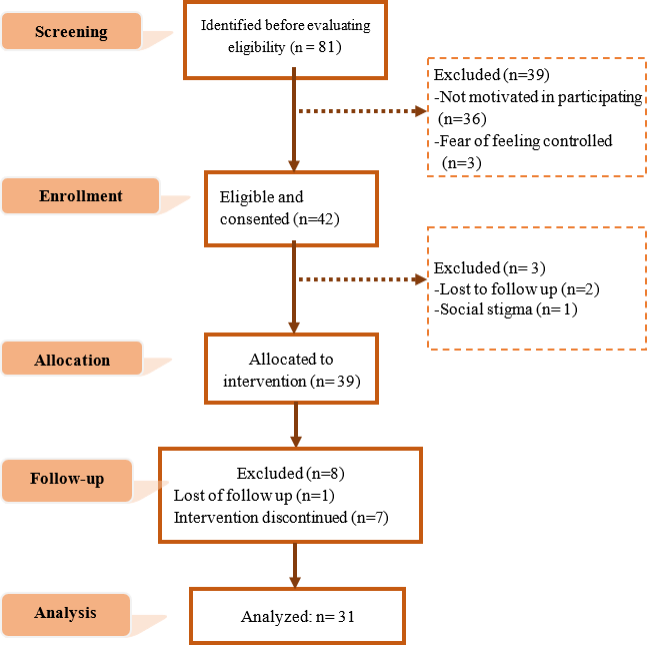
m-RESIST study participation flowchart. m-RESIST: mobile therapeutic attention for treatment-resistant schizophrenia.

The study participation flowchart based on the CONSORT (Consolidated Standards of Reporting Trials) 2010 statement recommendations [73] is presented in Figure 1.

### Background Variables

The sociodemographic characteristics of this study’s population (n=31) are presented in Table 2. No statistically significant differences in sociodemographic characteristics were found at baseline between the participants in the 3 sites. The percentage of men and women was almost equal (n=16, 51.6% and n=15, 48.4%, respectively). Most participants were singles (n=25, 80.6%) and lived with parents or relatives (n=23, 74.2%). Almost half of the study population had a full-time or a half-time job (n=15, 48.4%). The most common education level was primary studies (n=15, 48.4%). Almost two of the third lives with parents or relatives (n=20, 64.5%). Nearly all study participants had a caregiver (n=28, 90.3%; Table 2). Those patients without a caregiver had support in their immediate environment.

Nearly all participants were currently on 1 or 2 antipsychotics (30/31, 96%), and clozapine was the most used drug (11/31, 35.5%). Olanzapine, clozapine, and risperidone were previously the 3 most frequently taken antipsychotics (13/31, 41.9%; 9/31, 29%; and 9/31, 29%, respectively).

**Table 2.** The sociodemographic variables of this study’s population.

	Values
Age, mean (SD)	33.9 (7.6)
Gender, n (%)	
Male	16 (51.6)
Female	15 (48.4)
Marital status, n (%)	
Single	25 (80.6)
Married or with a stable partner	4 (12.9)
Divorced or separated	2 (6.5)
Cohabitation, n (%)	
Lives alone	4 (12.9)
Lives with parents or relatives	23 (74.2)
Lives with a stable partner	3 (9.7)
Lives with roommates	1 (3.2)
Occupation, n (%)	
Full-time job	7 (22.6)
Part-time job	8 (25.8)
Unemployed	9 (29)
Pension	6 (19.4)
Protected job	1 (3.2)
Educational level, n (%)	
Primary studies	15 (48.4)
Secondary studies	10 (32.3)
University	6 (19.4)
Lives with parents or relatives^a^, n (%)	
No	8 (25.8)
Yes	20 (64.5)
Caregiver availability^a^, n (%)	
No	2 (6.5)
Yes	28 (90.3)

a Missing n=1.

### Clinical Outcomes

Significant reductions were found in symptoms from pretrial to posttrial on the PANSS total (mean 73.7, SD 14.8 versus mean 66.5, SD 12.8; mean difference −7.2, 95% CI −11.1 to −3.4; *P*=.001), PANSS positive (mean 15.2, SD 2.8 versus 13.8, SD 3.2; mean difference −1.36, 95% CI −2.6 to −0.1; *P*=.04), PANSS negative (mean 21.2, SD 4.9 versus 19.1, SD 4.3; mean difference −2.1, 95% CI −3.1 to −1.1; *P*<.001), and PANSS general symptoms (mean 37.3, SD 9.3 versus 33.6, SD 8.4; mean difference 3.8, 95% CI −6.8 to −0.8; *P*=.02) (Table 3).

In almost one-fifth of the participants (6/31), the overall score for the PANSS decreased by more than 20%.

The sum of global severity of illness also decreased significantly on the CGI-SCH scale (median 4, IQR 3-4 vs median 3, IQR 3-4; median difference 0, IQR −1 to 0; *P=.*03). A decrease in the sum of positive (median 3, IQR 2.75-4 vs median 3, IQR 2-3.25; median difference 0, IQR −1 to 0; *P=.*04) and negative (median 4, IQR 3-4 vs median 3, IQR 3-4; median difference 0, IQR −1 to 0; *P=.*03) symptoms was also found. The sum of depressive or cognitive symptoms did not change (Table 3).

**Table 3.** Change of symptoms between baseline and follow-up.

		Baseline	Follow-up	Difference	*P* value
PANSS^a^^,b^					
	Total score	73.7 (14.8)	66.5 (12.8)	−7.2 (−11.1 to −3.4)	.001
	Positive symptoms score	15.2 (2.8)	13.8 (3.2)	−1.36 (−2.6 to −0.1)	.04
	Negative symptoms score	21.2 (4.9)	19.1 (4.3)	−2.1 (−3.1 to −1.1)	<.001
	Generalized symptoms score	37.3 (9.3)	33.6 (8.4)	−3.8 (−6.8 to −0.8)	.02
CGI-SCH^c^^,d^					
	Sum of positive symptoms	3 (2.75 to 4)	3 (2 to 3.25)	0 (−1 to 0)	.03
	Sum of negative symptoms	4 (3 to 4)	3 (3 to 4)	0 (−1 to 0)	.03
	Sum of depressive symptoms	3 (2 to 3.25)	3 (2 to 3)	0 (−1 to 0.5)	.22
	Sum of cognitive symptoms	3 (2.75 to 4)	3 (2 to 3)	0 (−1 to 0)	.23
	Sum of global severity	4 (3 to 4)	3 (3 to 4)	0 (−1 to 0)	.03
GAF^d^^,e^		55 (45 to 55)	60 (50 to 65)	5 (0 to 10)	<.001
EQ-VAS^b^^,f^		66.1 (18)	72.8 (16.5)	6.7 (0.5 to 12.9)	.04

a PANSS: Positive and Negative Syndrome Scale.

b Mean (SD) for the baseline and follow-up, and mean (95% CI) with paired samples *t*-test for the difference.

c CGI-SCH: Clinical Global Impression-Schizophrenia.

d Median (IQR) for the baseline and follow-up, and median (IQR) with Wilcoxon test for the difference.

e GAF: Global Assessment of Functioning.

f EQ-VAS: EuroQol visual analogue scale.

### Functional and Perceived Quality of Life Outcomes

The functionality of participants increased significantly from pretrial to posttrial on the GAF scale (median 55, IQR 45-55 vs median 60, IQR 50-65; median difference 5, IQR 0-10; *P*<.001), but it was not considered clinically significant due to participants remaining in the same interval (moderate symptoms).

The perceived quality of life evaluated by the EQ-VAS also improved (mean 66.1, SD 18 vs mean 72.8, SD 16.5; difference 6.7, 95% CI 0.5-12.9; *P=.*04) (Table 3).

### Adverse Events

The symptoms of TRS got worse in 1 participant before the intervention and in 2 participants during it, which was considered not to be related to the m-RESIST solution.

## Discussion

### Principal Results

To our best knowledge, this was the first study collecting data on the symptoms, psychosocial functioning, and quality of life for people with TRS by using the mHealth platform (m-RESIST solution). Our novel preliminary findings showed that the total, positive, negative, and general symptoms according to the PANSS were significantly decreased after intervention compared with the starting point. In almost one-fifth of the participants (6/31), the overall score for the PANSS decreased by more than 20%, which may be considered a clinically significant change [74].

According to Suzuki et al [75], the partial response of clinical trials in schizophrenia could be defined ≥10% to <20% decrease on the PANSS. In our study, the change in positive score was 8.9%. Interestingly, the change in the negative score was 10.2%, which may mean a partial response according to Suzuki`s definition. The decrease in the generalized symptoms scores and the total score was 10.2% versus 9.8%. Therefore, the mean differences of 1‐2 points on the positive and negative subscale scores may be indicatively clinically meaningful to a small extent at the most. Nevertheless, one should be careful in interpretations regarding the significance of the results. The clinical implications of PANSS scores may not always be clear [76].

The sum of global severity, and the sum of positive and negative symptoms on the CGI-SCH scale were lower at the end of the study compared to the starting point, but the sum of depressive or cognitive symptoms did not change. The functionality measured by the GAF scale was also significantly better at the end of this study as compared to its level at the beginning. The perceived quality of life also increased during this study.

### Comparisons With Prior Work

The finding of a significant decrease in all PANSS scores and global severity, positive and negative symptoms on the CGI-SCH scale at the study end compared to the starting point is partly in line with former data, as previous studies have shown efficacy of mobile interventions for schizophrenia [6,15,27,36,43,56,61,62]. To the best of our knowledge, this is the first study to show the efficacy of an mHealth platform (m-RESIST solution) in TRS.

A study by Ben-Zeev et al [6] demonstrated the feasibility, acceptability, and efficacy of a smartphone intervention for schizophrenia, followed by those with schizophrenia or schizoaffective disorder (N=33) for 1 month without a control group. Its results showed a significant decrease in the total, positive, and general scores but not in the negative score in the PANSS. A noncontrolled study by Kidd et al [25] with patients with primary psychosis (N=38) reported a decrease in certain items of the Brief Symptom Inventory (eg, psychotism, paranoid ideation, and depression) when followed for 1 month.

Bucci et al [27] carried out an RCT among those with early psychosis (N=36). The results showed a decrease in the total, negative, and general scores of the PANSS after a follow-up of 12 weeks.

WeChat is the most widely and extensively used mobile social networking app in China. A cross-sectional study on WeChat use and endorsement of WeChat-based mHealth among people living with schizophrenia (n=400) in China was carried out. Compared to nonusers in this study’s population, WeChat use was associated with improved health outcomes, including lower psychiatric symptoms (the Brief Psychiatric Rating Scale), lower depression (the Patient Health Questionnaire-9), higher functioning (GAF), better recovery (the Recovery Assessment Scale), and higher quality of life (the first 2 general questions from the World Health Organization Quality of Life Brief Scale) [62].

However, another RCT with participants with recent-onset schizophrenia spectrum disorders (N=43) showed no changes in their positive or negative score of the PANSS or their functioning measured by the Role Functioning Scale. The quality of life measured by the interview-based Quality of Life Scale Abbreviated did not improve [36]. A recent study with patients with a diagnosis of schizophrenia (N=361), randomized to receive either SlowMo*,* a blended digital therapy targeting reasoning, or usual care, found also no significant difference between groups on the primary outcomes related to paranoia (the Green et al [77] Paranoid Thoughts Scale) at the primary end point at 24 weeks [33].

### Decrease in Symptoms

It should be taken into account that the decrease in symptoms in patients with TRS, characterized by the persistent presence of symptoms, would not be expected in such a short term. Following the considerations of this study by Ben-Zeev et al [6], the improvement in the PANSS scores obtained in this study of the m-RESIST solution should be evaluated prudently. In the case of patients with schizophrenia, in the framework of a study, a placebo effect of the offered intervention has been observed. One factor that would influence the improvement of symptoms would be all the care received for participating in a study. It could be considered that participation in a structured intervention program would promote a state of security, such that it would improve aspects such as the symptoms of the disorder itself [78,79].

However, the overall score for the PANSS decreased by more than 20% in almost one-fifth of the participants which may be considered a clinically significant change [74]. Furthermore, the negative symptoms on the PANSS decreased during a rather short period of 3 months. It may imply that somehow the m-RESIST solution may manage to activate patients.

### Perceived Quality of Life and Functioning

The perceived quality of life and functioning improved in our study, which is partly in line with the earlier studies, as a study by Schlosser et al [36] did not find positive effects on functioning and quality of life. Instead, the findings of Yu et al [62] showed improvement both in functioning and quality of life. Our findings on the improvement of quality of life may need to be treated with caution as the findings of the EQ-5D-5L scale have shown weak correlations with the objective measures among patients with schizophrenia [80].

### Study Timeline

The data collection period was in 2017. Although progress has been made, there is still room for improvement in mHealth apps for people with schizophrenia, with fewer than 10 apps specifically designed for this population available for public download [43,59]. The m-RESIST solution is a new, innovative mHealth tool developed for patients with TRS and their caregivers, offering modular and flexible functions. This study aimed to evaluate the effects of m-RESIST on clinical outcomes, functional outcomes, and perceived quality of life in individuals with TRS. To the best of our knowledge no previous studies have addressed these specific issues.

For the above reasons, our research results provide significant new information on the use of mobile applications in the treatment of TRS.

### Strengths

This study has several strengths. To our best knowledge, this was the first study collecting data on the symptoms, functioning, and perceived quality of life among patients with TRS by an mHealth intervention. This was also a multicenter study covering different cultural environments. The m-RESIST solution is an innovative mHealth solution based on novel technology, offering highly modular and flexible functioning and planned intervention. It has been developed specifically for patients with TRS [63-65]. The outcomes of this study will also help to perform a cost-effectiveness RCT in the future.

### Limitations

Some limitations of this study need to be discussed. First, there was no control group, so it is not possible to determine whether the clinical improvements were related to the use of the m-RESIST solution. Second, this study’s design is a limitation, as, due to the small sample size and the short length of the intervention and follow-up period, the differences found in outcomes should be treated with caution. Therefore, the generalization and maintenance of the results are uncertain. Third, the so-called Hawthorne effect may also have implications for the generalizability of results because the behavior of research participants is influenced by observation and measurement. In general, results in experimental settings may be more positive compared to routine clinical applications [78,79].

The moderately high mean baseline score for the PANSS (73.7) demonstrates that our patients with TRS have quite severe psychotic symptoms. Even so, they responded to intervention markedly.

### Potential Selection Bias Regarding Digital Literacy

Digital literacy can be defined as the varying ability of both children and adults to use digital technologies and understand their risks. Patients who do not know how to use digital health tools, do not see the importance of those tools, or cannot access them in their preferred language, ultimately will not use them [81]. This may cause potential selection bias because, for the reasons above, those people may not participate in the studies [81].

### Technical Implementation Challenges

The digital divide remains a barrier, with people with schizophrenia eager and able to obtain smartphones and learn new skills, but resources and training are often not provided. Expanding train-the-trainer and literacy programs such as the Digital Health Navigator initiative, which was specially designed to support people with psychosis, offers 1 tangible next step [42,43,53] However, a lagging infrastructure and skill base have limited the application of digital solutions in mental health care [82]. Additionally, evidence is needed on the effectiveness and appropriateness of digital health tools for patients who are marginalized and may lack access to digital health interventions [83].

The digital divide remains a barrier, with people with schizophrenia eager and able to obtain smartphones and learn new skills, but resources and training are often not provided [51]. Expanding train-the-trainer and literacy programs such as the Digital Health Navigator initiative, which was specially designed to support people with psychosis, offers 1 tangible next step [53,84].

### Cost Implications for Real-World Implementation

Despite modest effect sizes, the relatively low cost and high scalability of most of the interventions tested support their public health relevance [85]. A recent review on this topic found only 37 relevant studies published in middle- and lower-income countries between 2016 and 2020, with the majority reporting feasibility and accessibility outcomes instead of efficacy, cost-effectiveness, or implementation [86]. However, the costs of implications for real-world implementation may be a concern, and that is why it has been asked if mHealth tools can decrease hospital stays and encourage patients to use mobile apps in favor of reducing costs [61]. Digital health tools still hold a unique potential to extend access to care in middle- and lower-income countries, where there is less investment or infrastructure around mental health [86]. Smartphone use is common and rapidly expanding in those countries, and thus represents a promising tool to reduce the mental health gap [42].

### Conclusions

Our preliminary findings showed that implementing the m-RESIST solution may decrease the symptoms and severity of illness and improve the functionality and perceived quality of life among people with TRS. The change of symptoms on the PANSS scores may be indicatively clinically meaningful to a small extent at the most. Nevertheless, one should be careful in interpretations regarding the significance of the results.

Implementing the m-RESIST solution may be a promising tool to improve the treatment even in TRS, and to offer a new tool for its present treatment options, already in the near future. If successful, this would be a very important step forward as TRS is a major clinical challenge, with usually poor outcomes. Before being implemented in daily practice, these results should be corroborated by performing controlled clinical trials with people with TRS, comparing treatment as usual with the m-RESIST solution.

## Abbreviations

CGI-SCH: Clinical Global Impression-Schizophrenia
CONSORT: Consolidated Standards of Reporting Trials
EQ-VAS: EuroQol visual analogue scale
GAF: Global Assessment of Functioning
ICT: information and communication technology
m-RESIST: mobile therapeutic attention for treatment-resistant schizophrenia
mHealth: mobile health
PANSS: Positive and Negative Syndrome Scale
RCT: randomized controlled trial
TRS: treatment-resistant schizophrenia
